# A complication of cosmetic medical tourism

**DOI:** 10.1016/j.jdcr.2024.04.042

**Published:** 2024-05-10

**Authors:** Guy Feraru, Tamar Koren, Bibiana Chazan, Michael Ziv, Marina Landau, Roni P. Dodiuk-Gad

**Affiliations:** a Ruth and Bruce Rappaport Faculty of Medicine, Technion, Haifa, Israel; b Division of Dermatology, Emek Medical Center, Afula, Israel; c Division of Infectious Diseases Unit, Emek Medical Center, Afula, Israel; d Arena Dermatology, Herzliya, Israel; e Division of Dermatology, Department of Medicine, Sunnybrook Health Sciences Center, University of Toronto, Toronto, Ontario, Canada

**Keywords:** complications, cosmetic medical tourism, *Mycobacterium abscessus*, nontuberculous mycobacterium

## History

**Figure 1:**
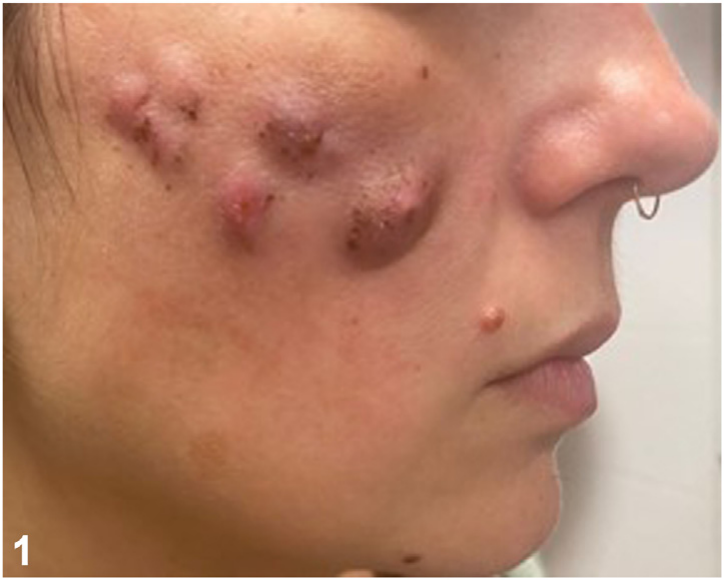

A 39-year-old woman, presented with a 1-month history of multiple, painful nodules on her cheeks, with no systemic manifestations (Fig 1). Her complaints started 4 days after injection of hyaluronic acid-based filler of an unknown brand, which took place during a vacation in a foreign country. She was initially treated by a family physician with amoxicillin-clavulanic acid followed by ciprofloxacin for 2 weeks, with no improvement. On physical examination, multiple erythematous, soft, and tender nodules were noted on the malar area of both cheeks, accompanied by nontender cervical lymphadenopathy. Routine laboratory tests were normal.

**Question 1: What is the most likely diagnosis?**

A: Injection site reaction
B: Acne fulminans
C: Acute facial cellulitis
D: Delayed inflammatory reaction to dermal filler
E: *Mycobacterium abscessus* infection

**Answers:**

**A:** Injection site reaction – Incorrect. Injection site reactions, such as erythema, swelling, pain, and bruising, are common immediate reactions, which generally resolve spontaneously within a week.
**B:** Acne fulminans – Incorrect. Acne fulminans is a rare disorder presenting as an acute, painful, ulcerating, and hemorrhagic lesions on face and trunk. It usually affects young males. It may be associated with systemic symptoms such as fever, polyarthritis, hepatosplenomegaly, leukocytosis, etc. X-rays may show osteolytic bone lesions.
**C:** Acute facial cellulitis – Incorrect. Acute facial cellulitis is mostly unilateral acute infection, caused by *Group A Streptococci* or *Staphylococcus aureus*, and is usually accompanied by systemic symptoms. Routine laboratory tests may show leukocytosis. Fourteen days treatment with systemic amoxicillin-clavulanic acid followed by ciprofloxacin is expected to improve symptoms of this condition.
**D:** Delayed inflammatory reaction to dermal filler – Incorrect. Delayed inflammatory reactions are immune mediated reactions to dermal fillers. They appear weeks to years after injection of resorbable or permanent fillers. Such reactions are usually not accompanied by regional lymphadenopathy, neither start on day 4 after the injection.
**E:** *Mycobacterium abscessus* infection – Correct. Inflammatory nodules, presenting within 3 to 7 days after filler injection are suspected as infection. In spite *Staphylococci* and *Streptococci* are the most identified organisms, limited response to first-line antibiotics and lack of growth from routine bacterial culture should raise suspicion of nontuberculous mycobacteria. The most common nontuberculous mycobacteria species causing postcosmetic procedures skin infection are *M abscessus*, followed by *Mycobacterium chelonae* and *Mycobacterium fortuitum.*^1^

**Question 2: What are the most appropriate next steps in the management of this patient?**

A: Incision and drainage
B: Sending a routine bacterial culture followed by a short course of intravenous amoxicillin-clavulanic antibiotic
C: Sending a routine bacterial culture and a tissue culture followed by treatment initiation of clarithromycin
D: Hyaluronidase injection
E: Sending discharge material from the nodules for tissue culture including specific *M abscessus* culture media, and antibiotics susceptibility testing followed by treatment initiation of a broad-spectrum antibiotic

**Answers:**

**Figure 2:**
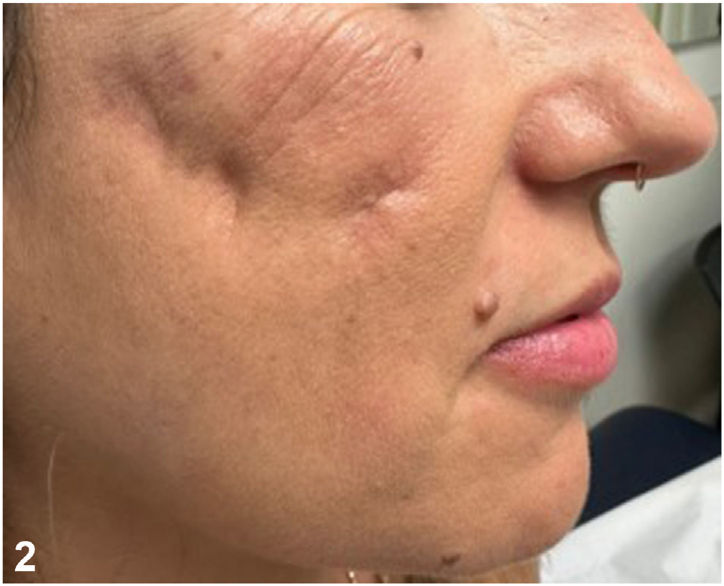

**Figure 3:**
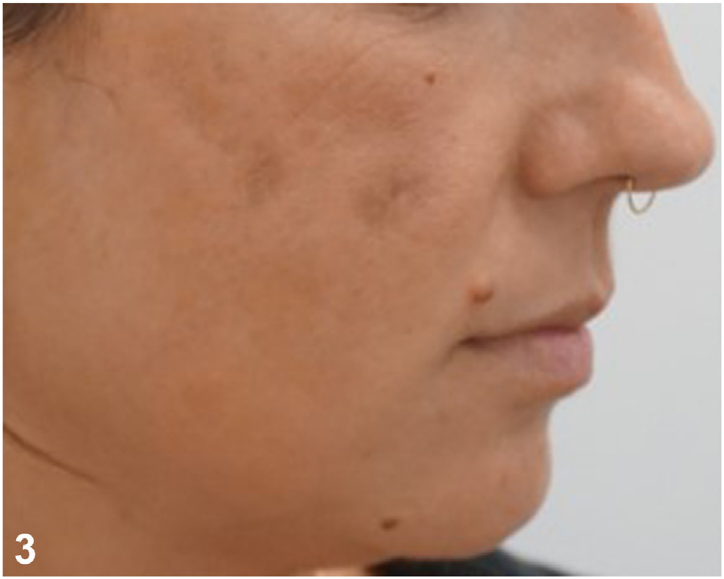

**A:** Incision and drainage – Incorrect. This rapid surgical procedure might offer an immediate pain relief but is insufficient to eradicate *M abscessus* soft tissue infection.
**B:** Sending a routine bacterial culture followed by a short course of intravenous amoxicillin-clavulanic antibiotic – Incorrect. Culture on specific growth media remains the gold standard, with *M abscessus* often growing within 7 days.
**C:** Sending a routine bacterial culture and a tissue culture followed by treatment initiation of clarithromycin – Incorrect. Antibiotic sensitivity testing is necessary since macrolide resistance is increasing among *M abscessus* isolates.^2^^,^^3^
**D:** Hyaluronidase injection – Incorrect. Although hyaluronidase is considered as a “gold standard” in treating delayed inflammatory reactions, it does not play a significant role in the treatment of filler induced infectious complications.
**E:** Sending discharge material from the nodules for tissue culture including specific *M abscessus* culture media, and antibiotics susceptibility testing followed by treatment initiation of a broad-spectrum antibiotic – Correct. In instances of skin infection following filler injections, clinicians should consider the potential involvement of nontuberculous mycobacteria. This necessitates thorough assessment through tissue cultures and antibiotic sensitivity testing. Following antimicrobial sensitivity results, our patient’s treatment was changed to oral clarithromycin and oral doxycycline. After 4 months of continuous antibiotic treatment all the nodules disappeared, but prominent atrophic scars associated with postinflammatory hyperpigmentation remained visible on both malar areas (Fig 2). These findings were treated by injection of hyaluronic acid-based filler and topical treatment with retinoic acid and hydroquinone with a substantial improvement (Fig 3).

**Question 3: Regarding cosmetic medical tourism – which of the next sentences is true?**

A: It refers to the practice of individuals traveling to locations outside the country of residence to undergo cosmetic procedures or surgeries
B: Injection of fillers is the primary source for cosmetic medical tourism related *M abscessus* infections
C: Cosmetic medical tourism has almost no drawbacks
D: Careful use of disinfecting agents such as chlorhexidine will be sufficed for *M abscessus* prevention
E: Medical tourism in general, should be discouraged, since it is unsafe practice

**Answers:**

**A:** It refers to the practice of individuals traveling to locations outside the country of residence to undergo cosmetic procedures or surgeries – Correct. Factors that contribute to the appeal to cosmetic medical tourism include cost saving, maintaining a level of privacy, and the allure of combining travel with treatment.
**B:** Injection of fillers is the primary source for cosmetic medical tourism related *M abscessus* infections – Incorrect. Mesotherapy is the most common procedure linked to *M abscessus* infections, followed by abdominoplasty, breast augmentation, liposuction, and filler injections.^1^
**C:** Cosmetic medical tourism has almost no drawbacks – Incorrect. This phenomenon has questionable quality assurance and lack of comprehensive postoperative care. Moreover, if complications occur after injection of a cosmetic product, no adequate information is usually available regarding the brand of the product and the injection technique that was applied.
**D:** Careful use of disinfecting agents such as chlorhexidine will be sufficed for *M abscessus* prevention – Incorrect. *M abscessus* possess a hydrophobic and lipid-rich cell, which facilitates biofilm formation on solid surfaces such as water pipes and medical implants. This attribute confers a degree of resistance to standard disinfectants including chlorine, organomercurials, alkaline glutaraldehyde, and 2% formaldehyde.^4^^,^^5^
**E:** Medical tourism in general, should be discouraged, since it is unsafe practice – Incorrect. Although traveling to “top of its kind” medical centers to treat severe or rare medical conditions or diseases is safely practiced for years, costs-driven cosmetic tourism is associated with increased health risks.

## Conflicts of interest

None disclosed.
